# In vitro combination effects and mechanisms of Revaprazan with Triazole antifungal drugs on *Aspergillus*

**DOI:** 10.1186/s12866-025-04471-w

**Published:** 2025-11-05

**Authors:** Wenxu Cheng, Min Shen, Lijia Wan, Tian Chen, Lingxi Wang, Heng Zhang, Yi Sun

**Affiliations:** 1 Department of Otolaryngology, Jingzhou Hospital Affiliated to Yangtze University, Jingzhou, Hubei 434020 China; 2 Hubei Provincial Clinical Research Center for Diagnosis and Therapeutics of Pathogenic Fungal Infections, Jingzhou, Hubei China; 3 Yangtze University, Jingzhou, Hubei 434020 China; 4 Department of Dermatology, Hubei Provincial Clinical Research Center for Diagnosis and Therapeutics of Pathogenic Fungal Infection Jingzhou, Jingzhou Hospital Affiliated to Yangtze University, Jingzhou, Hubei 434020 China

**Keywords:** Revaprazan hydrochloride, Acid pump antagonist, Azoles, MFS transporter

## Abstract

Potassium-competitive acid blockers (P-CABs), such as revaprazan (REV), act by competitively inhibiting potassium (K^+^) binding on the proton pump (H^+^/K^+^-ATPase) and are reversible K^+^ antagonists. This study evaluated REV combined with triazoles (itraconazole, ITR; voriconazole, VOR; posaconazole, POS) against 30 clinical *Aspergillus* isolates (14 *A. fumigatus*, 12 *A. flavus*, 4 *A. terreus*) using MIC and FICI assays. REV demonstrated dose-dependent synergy (FICI ≤ 0.5) with POS in 90% of isolates (27/30), reducing MIC values by 4–8fold, while synergy with ITR occurred in 20% (6/30). Species-specific analysis revealed highest efficacy against *A. flavus* (91.7%, 11/12) and *A. terreus* (100%, 4/4). No synergy was observed with VOR. Furthermore, the mechanisms underlying the synergistic effects of REV with triazoles were investigated using an *A. fumigatus* major facilitator superfamily (MFS) transporter gene-deficient strains. Knockout strains *ΔAF-MFS32* and *ΔAF-MFS35*, selected from our MFS transporter gene-deficient strains, showed a abolishment of synergistic interaction when combined with REV and the triazoles ITR and POS. Suggesting a functional link between these transporters and the drug interaction. Disk diffusion and efflux pump assays corroborated these findings (*P* < 0.05). These in vitro results identify REV + POS as a promising combination against *Aspergillus*, though in vivo studies and mechanistic validation of transporter inhibition are warranted.

Overall, this study shows that the potassium-competitive acid pump antagonist REV exhibits synergistic antifungal activity with triazoles in treating *Aspergillus* infections. The MFS transporter *AF-MFS32* and *AF-MFS35* are implicated as possible targets mediating the synergistic interaction between REV and these antifungal drugs.

**Supplementary Information:**

The online version contains supplementary material available at 10.1186/s12866-025-04471-w.

## Introduction

 The global prevalence of azole-resistant *A. fumigatus* has risen dramatically, with resistance rates exceeding 20% in regions with high agricultural or clinical azole use [1]. This trend severely limits treatment options, as azole failure in invasive aspergillosis increases mortality from 30% to nearly 90% [2]. In immunocompromised individuals, *Aspergillus* conidia can germinate into tissue-invasive hyphae, leading to the spread of invasive aspergillosis (IA) [3]. The treatment options for *Aspergillus* infections are limited, with triazoles being the mainstay of therapy. However, resistance to triazoles is increasingly reported [4]. The primary mechanisms of triazole resistance include: (1) mutations and overexpression of the cytochrome P450 sterol 14-α-demethylase gene (*cyp51A*) involved in the ergosterol synthesis pathway [5]; and (2) increased expression of efflux pump transporter proteins, such as ATP-binding cassette (ABC) transporters and major facilitator superfamily (MFS) transporters, which utilize proton-driven transport to efflux drugs [6].

Proton pump inhibitors (PPIs) are the first-line treatment for acid-related disorders due to their ability to inhibit H^+^/K^+^-ATPase on gastric parietal cells [7]. However, potassium-competitive acid blockers (P-CABs), such as revaprazan (REV), offer several advantages over conventional PPIs: (1) P-CABs achieve rapid therapeutic plasma levels and can almost completely suppress gastric acid secretion from the first dose; (2) P-CABs have minimal involvement in CYP2C19 metabolism, reducing drug interactions; (3) the effect of P-CABs is independent of the secretory state; and (4) P-CABs are stable in acidic environments without the need for enteric coating [8].

Previous studies have shown that PPIs, including pantoprazole, lansoprazole, omeprazole, and rabeprazole, act synergistically with triazoles to enhance their anti-*Aspergillus* activity [9]. Clinical reports suggest that combining PPIs with triazoles may improve outcomes in *Aspergillus* infections [10, 11]. However, PPIs carry risks of duodenal dysbiosis, pancreatitis, and NSAID-associated intestinal injury [12, 13], whereas P-CABs like REV avoid these limitations due to their distinct pharmacology [8].Notably, REV shares structural motifs with known efflux pump inhibitors, suggesting potential antifungal applications. Preliminary studies indicate P-CABs may modulate membrane transport systems in fungi [14], but their mechanisms remain uncharacterized.

Based on the critical role of MFS transporters in azole resistance and REV’s structural properties, we hypothesize that REV enhances triazole efficacy by specifically inhibiting *AF-MFS32* and *AF-MFS35* mediated efflux in *Aspergillus*. This study combines: (1) systematic evaluation of REV-azole synergy across 30 clinical isolates, and (2) mechanistic validation using MFS transporter knockout strains and rhodamine efflux assays.Thereby providing a theoretical basis for the use of REV as an antifungal sensitizer in treating patients with complicated or refractory *Aspergillus* infections.

## Materials and methods

### Fungal strains

In this study, a total of 30 clinical isolates were tested, comprising 14 *A. fumigatus* strains, 12 *Aspergillus flavus* strains, and 4 *Aspergillus terreus* strains. *Candida parapsilosis* (ATCC22019) and *A. flavus* (ATCC204304) were used as quality control strains. All clinical isolates were stored in our laboratory [9]. DNA was extracted from the isolates, and their internal transcribed spacer (ITS) regions were amplified and sequencing to confirm strain identity. All the sequences of the analyzed strains were uploaded to GenBank (PP069948-PP070390). *A. fumigatus* AF293, which contains the complete DNA sequence of *A. fumigatus*, was purchased from the Fungal Genetic Stock Center to serve as a template for amplifying all gene fragments. *A. fumigatus* A1160 (ΔKU80, *pyrG*-) was also sourced from the same center, but this strain lacks the pyrG gene and cannot synthesize uracil (U).In this study, it is used as the recipient strain for transformation. The *A. fumigatus* WT (ΔKU80, *pyrG+*) strain was obtained by randomly introducing the missing *pyrG* gene into *A. fumigatus* A1160,Since this strain does not lack the *pyrG* gene, it can successfully synthesize uracil and is used in this study as a control group. The plasmid pALX223 contains the *pyrG* gene, enabling it to autonomously synthesize U. If the knockout product is successfully introduced into the protoplast, new colonies will appear on a U-free medium, known as positive transformants, In this study, these positive transformants are used as screening markers to identify positive transformants. The *AF-MFS32* gene, GenBank serial number AFUA_1G03200, is located on chromosome 1 with five exons consisting of 1725 bases; the *AF-MFS35* gene, GenBank serial number AFUA_3G13520, is located on chromosome 3 with five exons consisting of 1759 bases. The genes *AF-MFS32* and *AF-MFS35* are MFS drug transporter protein-related coding genes.

### Antifungal agents

The antifungal agents used in this study were itraconazole (ITR; purity 99%; S2476), voriconazole (VOR; purity 99%; S1442), and posaconazole (POS; purity 99%; S1257), all of which were purchased from MedChemExpress (MCE). The working concentration ranges were 0.03–8 µg/mL for ITR and VOR, and 0. 015–4 µg/mL for POS. Revaprazan (REV; purity 99%; number 144887) was obtained from MCE China Haoyuan Biological Co. Ltd. with a working concentration range of 0.125–16 µg/mL.

### Inoculum preparation

Activated strains were inoculated on Sabouraud dextrose agar (SDA) plates and cultured for 3 days at 37 °C. Fungal conidia were harvested and adjusted to a concentration of 3 × 10^6^/mL using a hemocytometer. The conidial suspension was then diluted in RPMI-1640 medium to a final concentration of 3 × 10^4^/mL. RPMI-1640 was buffered with 3-(N-morpholino)propanesulfonic acid (MOPS) [15, 16].

### Checkerboard test

The checkerboard test was conducted following the microdilution checkerboard method as described in the CLSI M27-A3 [17] and M38-A2 [18] guidelines. Antifungal agents were diluted in RPMI-1640 to twice their highest final concentrations for subsequent serial dilutions. ITR and VOR were diluted in the range of 0.03–8 µg/mL, and POS was diluted in the range of 0.015–4 µg/mL, each in eight serial two-fold dilutions, with 50 µL per well. REV was diluted to a working concentration range of 0.5–16 µg/mL, also using eight gradients, with 50 µL per well. Column 1 was designated for REV alone as a single drug control, and row H was designated for each triazole as a single drug control. Subsequently, 100 µL of a two-fold concentration of the fungal suspension was added to each well of a 96-well plate and incubated at 35 °C for 48 h. Positive (growth) and negative (sterile) controls were included, along with wild-type and knockout strain controls. The minimum inhibitory concentration (MIC) was defined as the lowest concentration that completely inhibited visible fungal growth. MIC values for each drug, both alone and in combination, were determined. The fractional inhibitory concentration index (FICI) was calculated as previous described [19]. The formula for FICI is: FICI = (Ac/Aa) + (Bc/Bb), where Ac and Bc are the MICs when used in combination, and Aa and Bb are the MICs when used alone.The interactions between the two drugs were classified as synergistic (FICI ≤ 0.5), indifferent (0.5 < FICI ≤ 4), or antagonistic (FICI >4) [20].

### Construction of knockout strains

The targeted knockout was accomplished using the previously described fusion PCR cassette and protoplast-based transformation approach [21, 22], with the target gene being replaced by the *pyrG* gene in the pLAX223. Three distinct DNA pieces were amplified from AF293, each including a 1.2 kb region upstream and downstream of target gene coding sequence and a *pyrG* cassette as the selectable marker (Primers for amplification were seen in Table S1). The amplification conditions are shown in Table S1. Fusion PCR conditions were utilized as described [23, 24]. A major band at 4.0 kb was shown after confirmation of the fusion PCR product by agarose gel electrophoresis. The polyethylene glycol (PEG)-mediated protoplast method was used for cassette transformation. The validation products were subjected to next-generation sequencing (Sangon Biotech, Shanghai, China) to confirm the success of knockout strains construct.

### Disk diffusion method

The disk diffusion method was performed following the CLSI M44-A2 protocol [25]. Colonies of activated strains grown on SDA plates at 35 °C were collected with a sterile cotton swab and suspended in 1 mL of sterile water in Eppendorf tubes. The spore concentration was adjusted to 1–5 × 10^6^/mL. The suspension was evenly spread on RPMI-1640 agar plates and allowed to dry. Sterile 8 mm diameter filter paper disks were placed at equal intervals on the agar surface. REV and POS, either alone or in combination, were applied at a concentration of 8 µg/mL (2 µL per disk). A control group was also included. After drying, the plates were incubated at 35 °C for 48 h, and the diameter of the inhibition zones was measured. This experiment was repeated three times on different days to ensure reproducibility. The data were calculated by multiple t-test to calculate the P value. Compared with the control group WT, the inhibitory effect of REV and POS combined on *ΔAF-MFS32*, *ΔAF-MFS35* and WT was evaluated.

### Rhodamine 6G-Active determination of external discharge pump

Follow the steps below: Cultivate spores on SAB solid medium for 24 h. Collect the spores using PBS buffer and wash them twice by centrifugation (3000xg, 5 min, 20 °C) to remove any residual medium. Resuspend the spores in HEPES-NaOH buffer (50mM; pH 7.0) and adjust the final concentration to 1 × 10^6^/mL. Add 2-deoxyglucose (5mM) and rhodamine 6G (10µM) to the cell suspension. Incubate at 30 °C, 200 rpm for 90 min to allow cells to accumulate rhodamine under glucose starvation conditions. Wash the starved cells twice in HEPES-NaOH buffer to remove unbound rhodamine 6G. Transfer 400 µl of the washed cell suspension to a 96-well flat-bottom microplate and incubate at 30 °C for 5 min. Add glucose (2mM) to initiate the efflux of rhodamine. Centrifuge the cells at specified time intervals (0, 5, 10,15,20,30 min) after glucose addition. Transfer one-third of the 100 µl cell supernatant to each well of the 96-well flat-bottom microplate. Measure the rhodamine fluorescence of the samples using a microplate reader with an excitation wavelength of 529 nm and an emission wavelength of approximately 553 nm.

### Pharmacological dissection of rhodamine 6G efflux in *ΔAF-MFS32* and *ΔAF-MFS35* knockout strains

To validate the role of MFS transporters in Rhodamine 6G (Rh6G) efflux, the proton ionophore CCCP was employed to dissipate the proton motive force (PMF), thereby specifically inhibiting MFS-mediated transport. After culturing spores on SAB solid medium for 24 h, they were harvested and washed twice with PBS buffer via centrifugation (3000 × g, 5 min, 20 °C) to remove residual medium. Cells in the logarithmic growth phase were washed and subsequently treated for 15 min at 37 °C in the dark under one of the following conditions: 20 µM CCCP (experimental group), an equivalent volume of DMSO (solvent control), or a combination of CCCP and 50 µM sodium vanadate (an ABC transporter inhibitor; positive control). Following pretreatment, 2-deoxyglucose (5 mM) and Rh6G (10 µM) were added to the cell suspensions, which were then incubated for 90 min at 30 °C with shaking at 200 rpm to allow Rh6G accumulation under glucose starvation. The starved cells were washed twice with HEPES-NaOH buffer to remove unbound Rh6G. Washed cell suspensions (400 µl) were transferred to a 96-well flat-bottom microplate and pre-incubated for 5 min at 30 °C. Glucose (2 mM) was then added to initiate Rh6G efflux. At specified time intervals after glucose addition (0, 5, 10, 15, 20, and 30 min), cells were centrifuged. One-third of the resulting supernatant (100 µl) from each sample was transferred to individual wells of a new 96-well plate. Rh6G fluorescence was measured using a microplate reader with excitation and emission wavelengths set at 529 nm and 553 nm, respectively. The contribution of MFS transporters was assessed by comparing efflux activity between the CCCP-treated and DMSO control groups, while ABC transporter activity was evaluated by comparing the CCCP group with the CCCP plus vanadate group.

## Results

### In vitro antifungal activities of the tested drugs

The MIC values of the tested drugs used to treat the *Aspergillus* isolates were ≥ 16 µg/mL for REV, 0.5–4 µg/mL for ITR, 0.5–1 µg/mL for VOR, and 0.5–1 µg/mL for POS (Table 1).

**Table 1:** Results of MIC and FICI for the combination of REV and antifungal drugs against *Aspergillus* strains

Strains	MIC alone (µg/mL)				MIC combinations (µg/mL)		
	REV	ITR	VOR	POS	REV/ITR	REV/VOR	REV/POS
AF1	> 16	2	0.5	0.5	0.25/2(I)	0.25/1(I)	8/0.125(S)
AF2	> 16	4	0.5	1	0.25/4(I)	0.25/1(I)	8/0.25(S)
AF3	> 16	2	1	1	0.25/2(I)	0.25/1(I)	4/0.25(S)
AF4	> 16	2	1	0.5	0.25/2(I)	0.25/1(I)	8/0.125(S)
AF5	> 16	2	0.5	1	0.25/2(I)	0.25/1(I)	8/0.25(S)
AF6	> 16	4	1	1	0.25/4(I)	0.25/0.5(I)	8/0.25(S)
AF7	> 16	2	0.5	1	0.25/2(I)	0.25/0.5(I)	8/0.25(S)
AF8	> 16	4	1	1	0.25/4(I)	0.25/1(I)	0.25/1(I)
AF9	> 16	2	0.5	0.5	8/0.5(S)	0.25/1(I)	8/0.125(S)
AF10	> 16	2	0.5	0.5	4/0.5(S)	0.25/1(I)	8/0.125(S)
AF11	> 16	4	0.5	1	0.25/4(I)	0.25/0.5(I)	8/0.25(S)
AF12	> 16	1	0.5	0.5	0.25/1(I)	0.25/0.5(I)	4/0.125(S)
AF13	> 16	2	1	0.5	8/8(A)	0.25/1(I)	0.25/0.5(I)
AF14	> 16	1	0.5	0.5	0.25/1(I)	0.25/0.5(I)	2/0.125(S)
AFLA1	> 16	2	0.5	0.5	0.25/2(I)	0.25/0.5(I)	4/0.25(S)
AFLA2	> 16	2	1	0.5	0.25/2(I)	0.25/0.5(I)	8/0.125(S)
AFLA3	> 16	2	1	0.5	0.25/2(I)	0.25/0.5(I)	8/0.125(S)
AFLA4	> 16	2	1	1	0.25/2(I)	0.25/0.5(I)	8/0.25(S)
AFLA5	> 16	1	1	1	0.25/1(I)	0.25/1(I)	8/0.25(S)
AFLA6	> 16	1	1	1	0.25/1(I)	0.25/1(I)	8/0.25(S)
AFLA7	> 16	0.5	1	0.5	0.25/0.5(I)	0.25/1(I)	2/0.125(S)
AFLA8	> 16	0.5	0.5	0.25	0.25/0.5(I)	0.25/0.5(I)	4/0.0625(S)
AFLA9	> 16	1	0.5	0.5	0.25/1(I)	0.25/1(I)	0.25/0.5(I)
AFLA10	> 16	1	1	1	0.25/1(I)	0.25/1(I)	8/0.25(S)
AFLA11	> 16	2	1	1	4/0.5(S)	0.25/0.5(I)	8/0.25(S)
AFLA12	> 16	1	0.5	1	2/0.5(I)	0.25/1(I)	2/0.25(S)
AT1	> 16	1	0.5	1	8/0.25(S)	0.25/0.5(I)	8/0.25(S)
AT2	> 16	2	0.5	0.5	8/0.5(S)	0.25/0.5(I)	4/0.125(S)
AT3	> 16	2	0.5	0.5	8/0.5(S)	0.25/0.5(I)	8/0.125(S)
AT4	> 16	1	0.5	0.5	0.25/1(I)	0.25/0.5(I)	4/0.125(S)
ATCC22019	> 16	0.5	0.125	0.25	0.25/0.5(I)	0.25/0.125(I)	4/0.0625(S)
ATCC204304	> 16	2	0.5	1	0.25/2(I)	0.25/0.5(I)	8/0.5(I)

*AF* *A. fumigatus* strains, *AFLA* *A. flavus*; *AT* *A. terreus*, *ITR *Itraconazole, *VOR *Voriconazole, *POS *Posaconazole, *REV *Revaprazan, *S *Synergy (FICI ≤ 0.5); I, indifference (no interaction, FICI from > 0.5 to ≤ 4). MICs were the concentrations that achieved 100% growth inhibition

### In vitro interactions of REV and the antifungal agents

When REV and ITR were combined, the MIC values of these two drugs against the clinical isolates decreased to 0.25–8 µg/mL and 0.25–4 µg/mL, The drug combination exhibited synergistic effects (FICI ≤ 0.5) against 20% of the evaluated *Aspergillus* isolates, comprising 2 *A. fumigatus* strains, 1 *A. flavus* strains, and 3 *A. terreus* strains (Tables 1 and 2).

**Table 2:** Summary of drug interactions for the combination of REV and antifungal agents

Species (*n*)	*n* (%) of isolates showing synergism for the combination		
	REV + ITR	REV + VOR	REV + POS
*A. fumigatus*(14)	2 (14.3)	0 (0)	12 (85.7)
*A. flavus*(12)	1((8.3)	0 (0)	11 (91.7)
*A. terreus(*4)	3 (75)	0 (0)	4 (100)
*Total*(30)	6 (20)	0 (0)	27 (90)

When REV and POS were combined, the MIC values of these two agents decreased to 0.25–8 µg/mL and 0.0625–1 µg/mL, The drug combination exhibited synergistic effects (FICI ≤ 0.5) against 90% of the evaluated evaluated *Aspergillus* strains, comprising 12 *A. fumigatus* strains, 11 *A. flavus* strains, and 4 *A. terreus* strains (Tables 1 and 2).

When REV and VOR were combined, the MIC values of these two antifungal agents decreased to 0.25 µg/mL and 0.125–1 µg/mL, respectively, and no synergistic or antagonistic effects on *Aspergillus* isolates were observed (Tables 1 and 2).

In the REV + POS alliance,*A. fumigatus*: 85.7% isolates (12/14) showed synergy;*A. flavus*: 91.7% isolates (11/12) showed synergy༛*A. terreus*: 100% isolates (4/4) showed synergy. POS consistently enhanced REV activity across all species, suggesting broad-spectrum potential.

In the REV + ITR alliance, *A. terreus*: 75% synergy (3/4 isolates), the highest among species.;*A. fumigatus*: 14.3% synergy (2/14 isolates)༛*A. flavus*: 8.3% synergy (1/12 isolates).ITR may have niche utility against *A. terreus* but limited synergy with REV for other species.

In the REV + VOR alliance, No synergy observed in any isolate (0/30 total); VOR is not a viable partner for REV in these *Aspergillus* species.

**Fig. 1:**
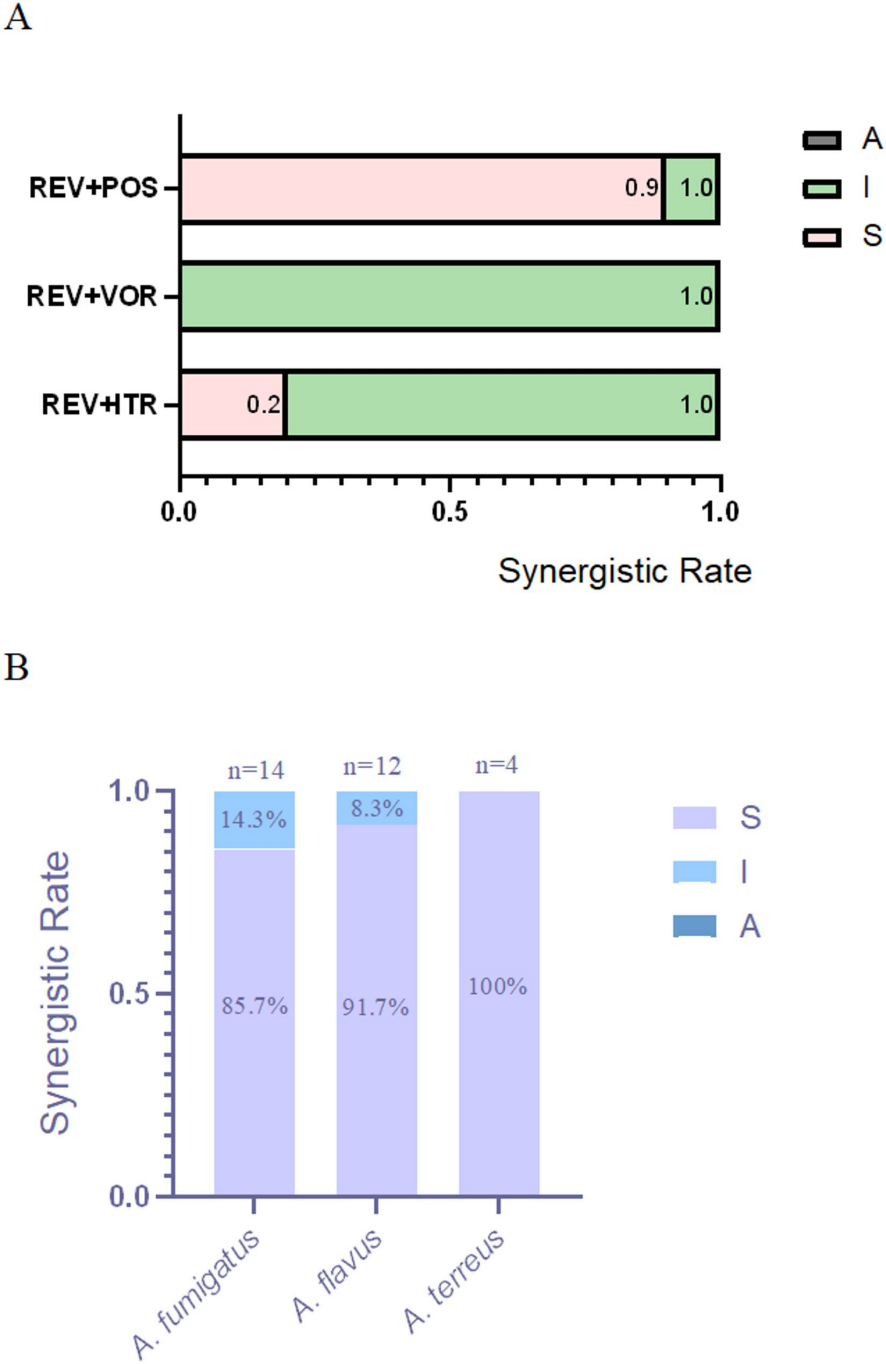
The Synergistic Rate of REV Combined with Azoles against *Aspergillus* spp. Note: **A**, interaction profile of REV combined with ITR, VOR and POS against *Aspergillus* spp; **B**, REV + POS interaction profiles across *Aspergillus* species

Thus, we conclude that: REV + POS was universally effective (85.7–100% synergy), but most potent against *A. flavus* (91.7%);REV + VOR failed in all cases, while REV + ITR showed sporadic activity (useful only for *A. terreus*).The synergy data is now explicitly compared by species, demonstrating that REV + POS is the most promising combination, particularly for *A. flavus*. The lack of synergy with VOR is also highlighted as a critical negative result.(Fig. 1).

### Construction of knockout strains for the genes encoding MFS transporters in *A. fumigatus*

**Fig. 2:**
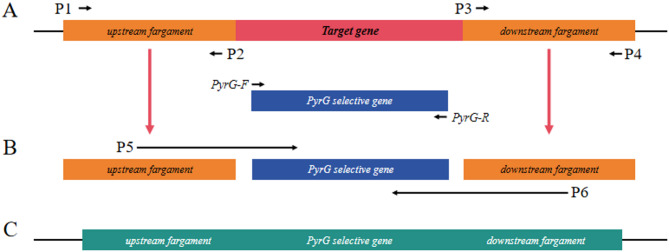
Schematic diagrams of primer binding sites and gene tissues for gene knockout and knockout verification. **A** Amplify the upstream and downstream DNA fragments of target geneand amplify the pyrG fragments labeled for screening. **B** Construct the fusion PCR products (UP, pyrG, DOWN). **C** Construct knockout confirmatory products to detect whether the gene has been successfully knocked out

**Fig. 3:**
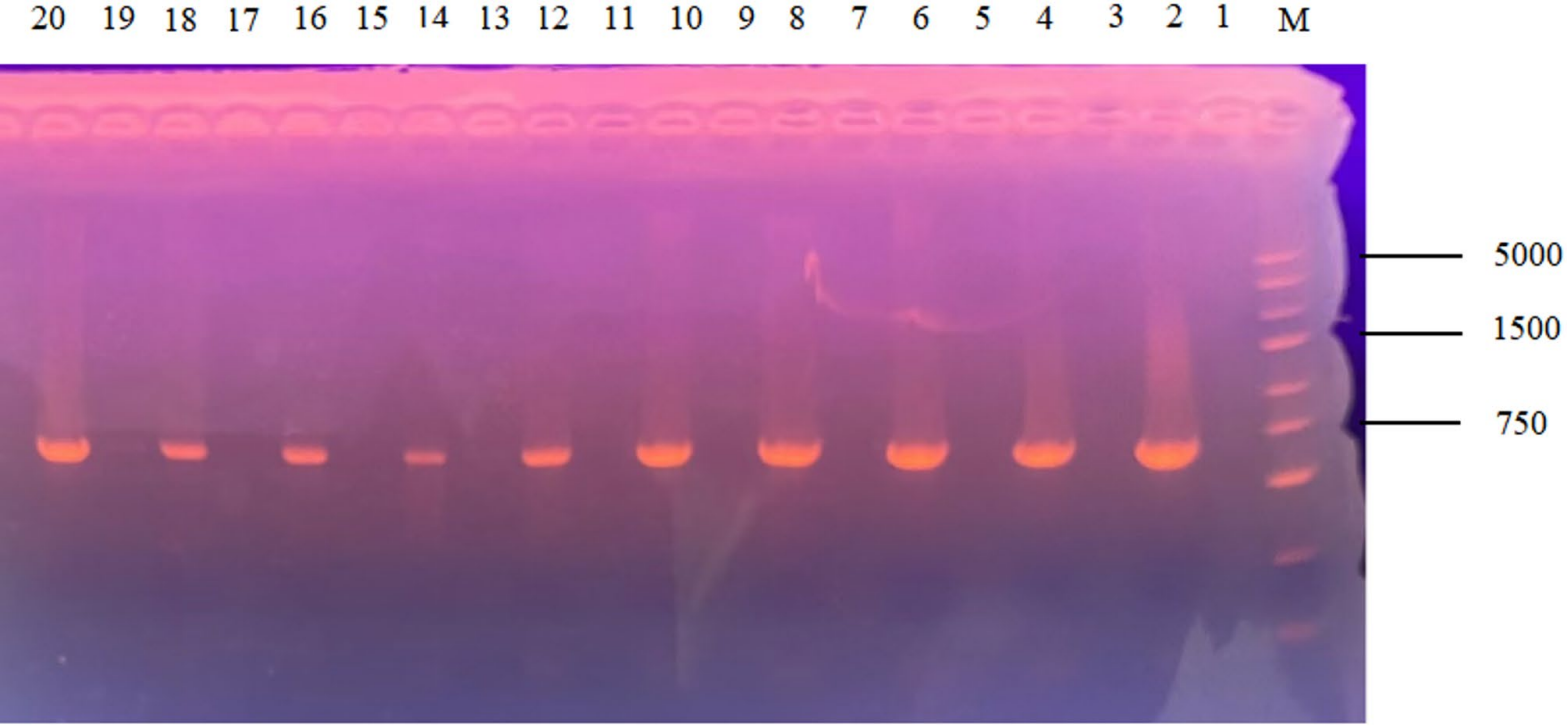
Electrophoretic gel for mutant confirmation. Note: M: DNA5000 Marker; 1, 3, 5, 7, 9, 11, 13, 15, 17, 19: No obvious bands were observed in the amplification of the target gene; 2, 4, 6, 8, 10, 12, 14, 16, 18, 20: Ten of these target genes corresponding to the pyrG fragment

**Fig. 4:**
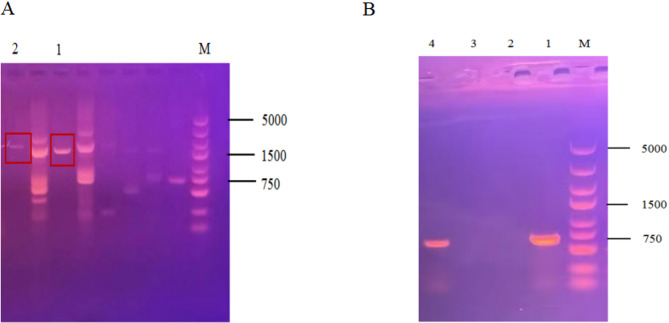
Verified electrophoretic gel diagram. Note: **A**: M: DNA5000 Marker; 1: The bands were amplified with primers P4 and Awm-F1 downstream of *AF-MFS32*; 2: The bands were amplified with primers P1 and Carslan-R4 upstream of *AF-MFS35*. **B**:1: The *AF-MFS32* *pyrG* fragment; 2: No obvious bands were observed in the amplification of the target gene; 3: No obvious bands were observed in the amplification of the target gene; 4: The *AF-MFS35* pyrG fragment

Twelve MFS transporter genes were selected by NCBI database, and 12 MFS knockout strains were successfully constructed by PCR validation and sequencing analysis. The knockdown construction process is shown in Fig 2. Electrophoresis showed that the relevant 12 target genes failed to amplify the bands, while the *pyrG* gene amplified clear bands, and the electrophoretic profiles of 10 target genes is shown in Fig 3. Target genes *AF-MFS32* and *AF-MFS35* are shown in Fig 4B; moreover, confirming successful knockout of *AF-MFS32* and *AF-MFS35*, electrophoresis analysis revealed 1.6 kb fragments amplified using *AF-MFS32* downstream primers P4 and Awm-F1 and 1.8 kb fragments using *AF-MFS35* upstream primers P1 and Carslan-R4 in Fig 4A.

### In vitro interaction of REV with antifungal agents in MFS transporter genes knockout strains

When REV, ITR, and VOR were combined, none of the 12 knockout strains of MFS transporter gene showed obvious synergistic activity. When REV and POS were combined, ten of these isolates demonstrated a synergistic activity. However, in the two knockout strains of *ΔAF-MFS32* and *ΔAF-MFS35*, the synergistic effect of REV and POS was reversed. (Table 3)

**Table 3:** Statistical table of antifungal sensitivity test

strain	MIC alone (µg/mL) MIC combinations (µg/mL)									
	REV	ITR	VOR	POS	REV/ITR	FICI	REV/VOR	FICI	REV/POS	FICI
WT(wild strain)	> 16	1	0.25	0.5	2/0.5(I)	1.016	0.25/0.25(I)	1.008	2/0.125(S)	0.313
*ΔAF-MFS13*	> 16	2	0.25	2	0.25/2(I)	1.008	0.25/0.25(I)	1.008	8/0.5(S)	0.5
*ΔAF-MFS14*	> 16	1	0.25	1	0.25/1(I)	1.008	0.25/0.25(I)	1.008	4/0.25(S)	0.375
*ΔAF-MFS15*	> 16	1	0.25	1	0.25/1(I)	1.008	0.25/0.25(I)	1.008	4/0.25(S)	0.375
*ΔAF-MFS24*	> 16	2	0.25	1	0.25/2(I)	1.008	0.25/0.25(I)	1.008	4/0.25(S)	0.375
*ΔAF-MFS26*	> 16	2	0.25	1	0.25/2(I)	1.008	0.25/0.25(I)	1.008	8/0.25(S)	0.5
*ΔAF-MFS27*	> 16	2	0.5	0.5	0.25/2(I)	1.008	0.25/0.5(I)	1.008	8/0.125(S)	0.5
*ΔAF-MFS32*	> 16	2	0.25	0.25	0.25/2(I)	1.008	0.25/0.25(I)	1.008	4/0.125(I)	0.625
*ΔAF-MFS35*	> 16	2	0.5	0.25	0.25/2(I)	1.008	0.25/0.5(I)	1.008	0.25/0.5(I)	2.008
*ΔAF-MFS42*	> 16	2	0.25	2	4/1(I)	0.625	0.25/0.25(I)	1.008	8/0.5(S)	0.5
*ΔAF-MFS47*	> 16	2	0.25	2	0.25/2(I)	1.008	0.25/0.25(I)	1.008	8/0.5(S)	0.5
*ΔAF-MFS58*	> 16	2	0.25	1	0.25/2(I)	1.008	0.25/0.25(I)	1.008	4/0.25(S)	0.375
*ΔAF-MFS67*	> 16	2	0.25	2	0.25/2(I)	1.008	0.25/0.25(I)	1.008	8/0.5(S)	0.5

*ITR *Itraconazole, *VOR *Voriconazole, *POS *Posaconazole, *REV *Revaprazan, *S *Synergy (FICI ≤ 0.5); I, indifference (no interaction, FICI from > 0.5 to ≤ 4). MICs were the concentrations that achieved 100% growth inhibition, *FICI *Fractional inhibitory concentration index

### Disk diffusion method

**Fig. 5:**
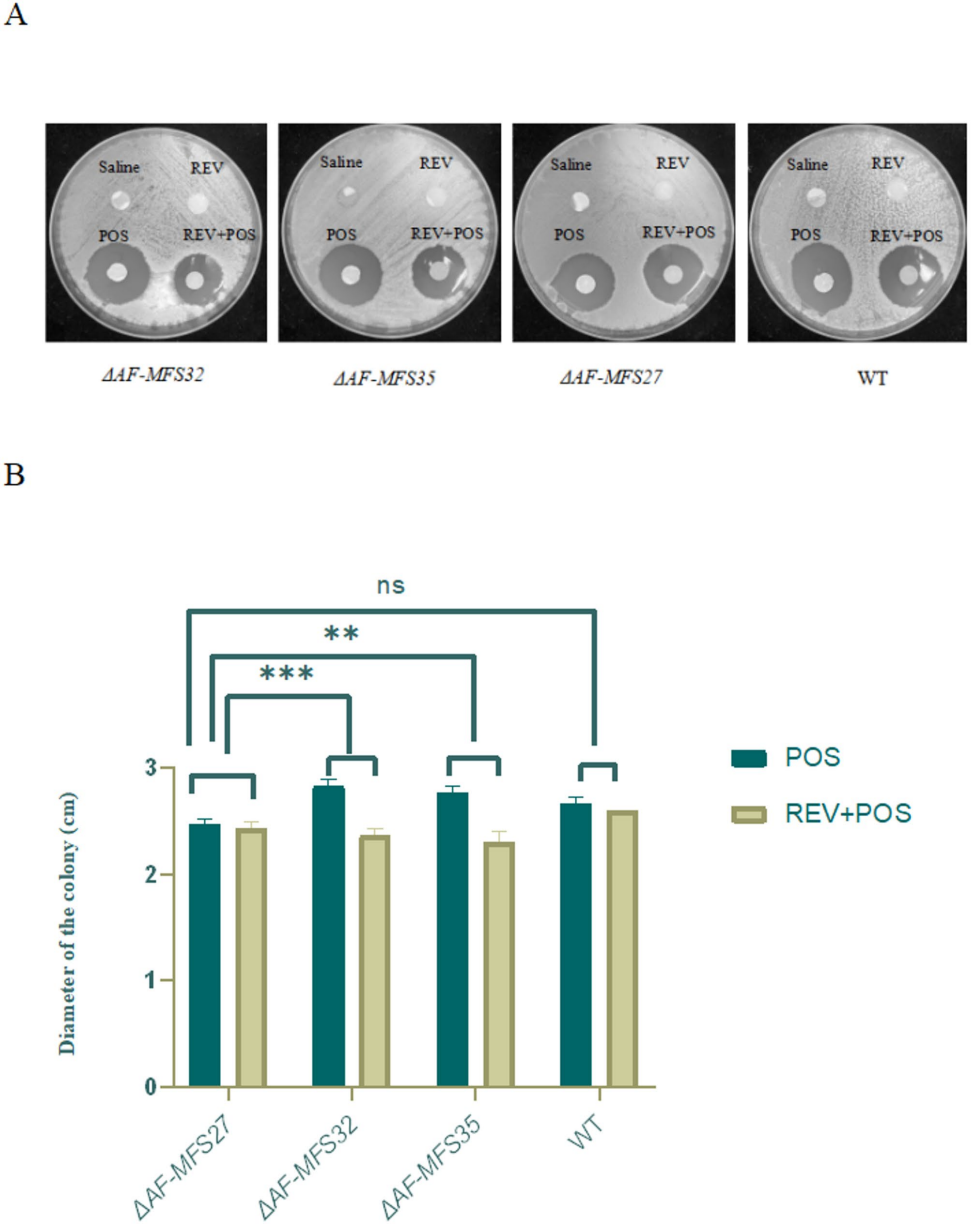
Results of disk diffusion method. Note: **A** When REV combined with POS, the inhibition zone of *ΔAF-MFS32*, *ΔAF-MFS35* was significantly smaller than that of control group *ΔAF-MFS27*. **B** REV: Revaprazan (8 µg/ml);POS: posaconazole (8 µg/ml). **P < 0.01,***P < 0.005

The combination of REV and POS exerted a significantly lower inhibitory effect on the *ΔAF-MFS32* and *ΔAF-MFS35* than on the control WT suggesting a statistically significant difference (*P* < 0. 05). (Fig 5)

### Rhodamine 6G - Efflux pump activity assay

**Fig. 6:**
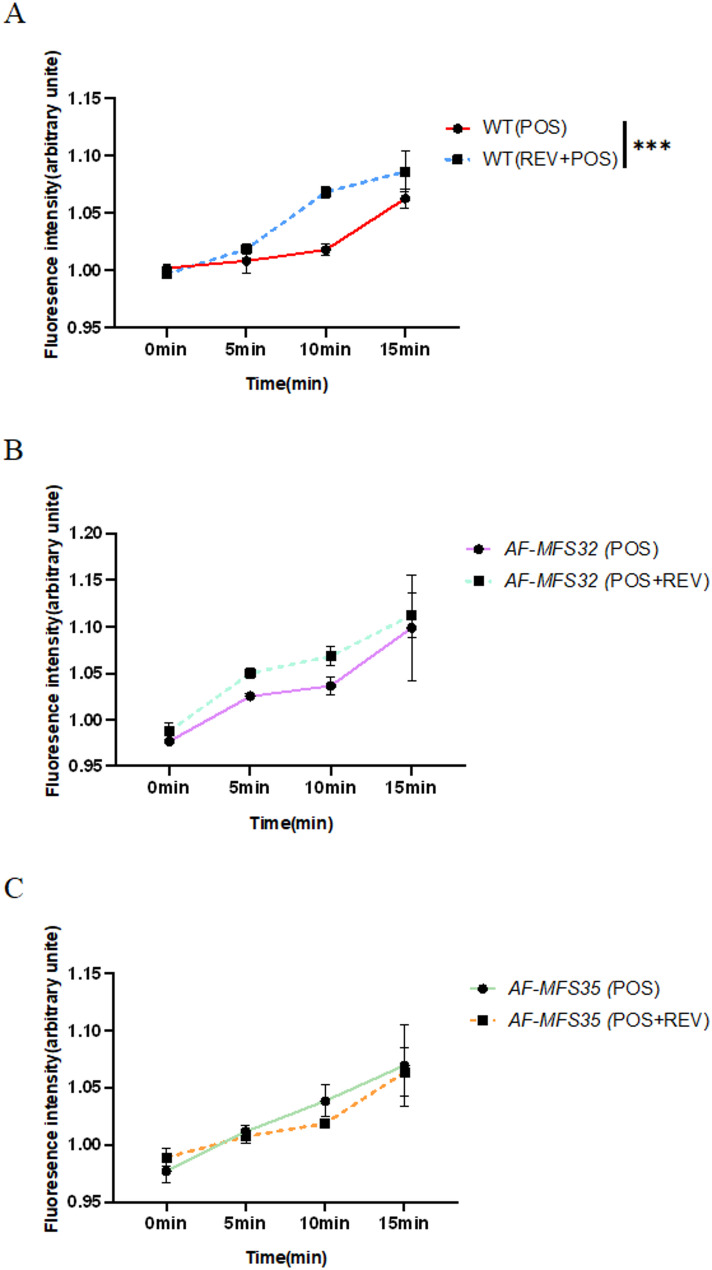
Rhodamine 6G efflux pump activity. Note: **A**: When the wild-type strain WT was used together with POS in REV and POS, the efflux pump activity was significantly enhanced compared with that of POS alone, which was statistically significant. **B** No statistically significant difference in efflux pump activity was observed in the *AF-MFS32* knockout strain between POS monotherapy and its combination with REV and POS. **C** No statistically significant difference in efflux pump activity was observed in the *AF-MFS35* knockout strain between POS monotherapy and its combination with REV and POS

The experiment to assess the efflux pump activity of knockout strains *ΔAF-MFS32*, *ΔAF-MFS35*, and the wild-type strain WT uses Rhodamine 6G as a substrate for the efflux pump. The accumulation and efflux of Rhodamine 6G reflect the pump’s function. According to the time-fluorescence curve, the efflux pump activity of the wild-type strain WT is significantly enhanced when REV and POS are used together compared to when POS is used alone, with a statistically significant difference. In contrast, the differences in efflux pump activity between the knockout strains *ΔAF-MFS32* and *ΔAF-MFS35* when POS is used alone and when REV and POS are used together are not statistically significant (Fig. 6).

### Pharmacological dissection of Rhodamine 6G efflux in *ΔAF-MFS32* and *ΔAF-MFS35* knockout strains

**Fig. 7:**
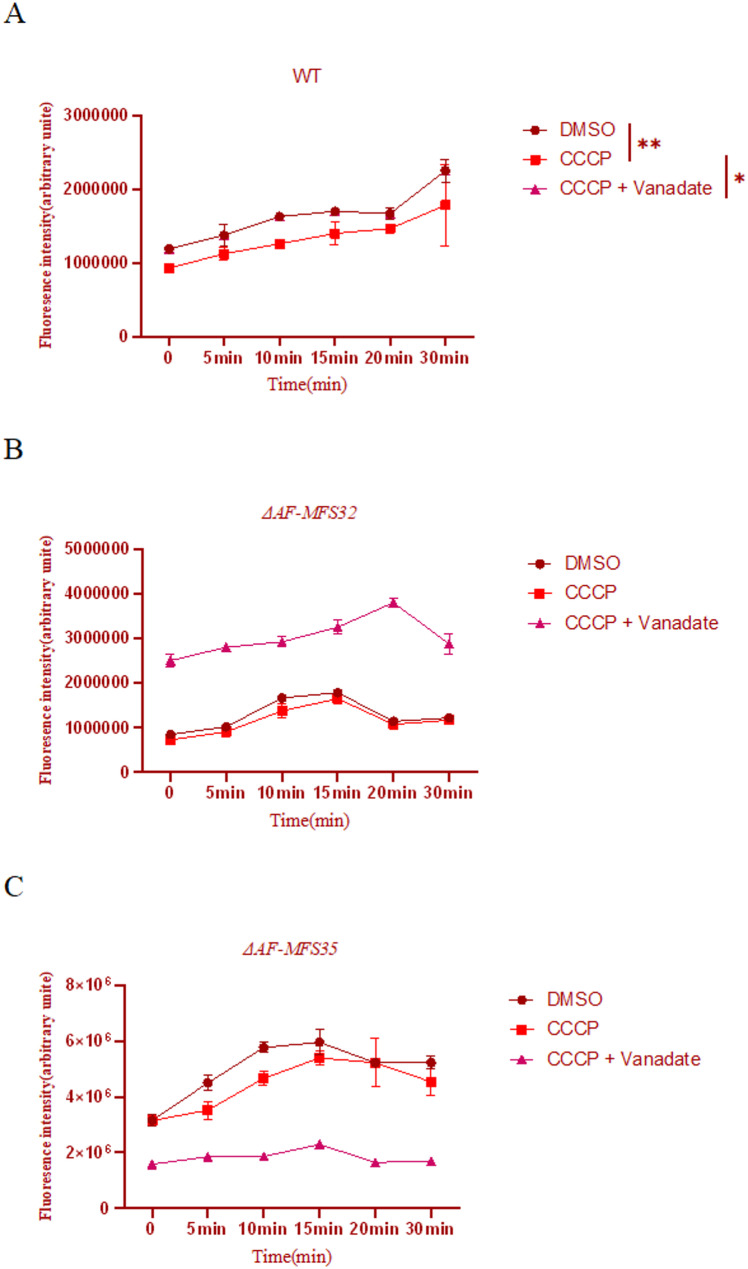
Pharmacological dissection of Rhodamine 6G efflux in *ΔAF-MFS32* and *ΔAF-MFS35* knockout strains. Note: (**A**-**C**) Time-course analysis of intracellular Rhodamine 6G (Rh6G) accumulation in thewild-type (WT, A), *ΔAF-MFS32* (B), and *ΔAF-MFS35 *(C) strains under different pharmacological treatments. Fluorescence intensity is inversely proportional to efflux pump activity. Treatments: DMSO (vehicle control), CCCP (20 µM, protonophore that collapses the proton motive force and inhibits MFS transporters), and CCCP + Vanadate (50 µM, ABC transporter inhibitor). Data are presented as mean ± SEM (*n* = 3).**P < 0.01;*P < 0.05

To unambiguously distinguish the types of transporters mediating Rh6G efflux, we further employed pharmacological inhibitors for validation. The experimental results (Fig. 7) demonstrated that in the WT strain, treatment with the proton ionophore CCCP (which abolishes the function of MFS transporters) resulted in a significant suppression of Rh6G efflux activity compared to the DMSO control group (*p* < 0.01). Subsequent addition of the ABC transporter inhibitor sodium vanadate led to a further significant inhibition of efflux activity (*p* < 0.05). This confirms that in the wild-type strain, Rh6G efflux is cooperatively mediated by MFS transporters (major contribution) and ABC transporters (minor contribution).

In contrast, both knockout strains, *ΔAF-MFS32* and *ΔAF-MFS35*, exhibited patterns strikingly different from that of the WT. No statistically significant differences were observed between the REV + POS combination treatments in either knockout (*p* > 0.05). More importantly, CCCP treatment failed to effectively inhibit the efflux activity in both knockout strains (*p* > 0.05 compared to their respective DMSO controls), and no significant difference was observed between the CCCP and CCCP + Vanadate treatments.

In the WT strain (A), CCCP treatment caused a dramatic increase in Rh6G accumulation (inhibition of efflux), indicating that efflux is primarily dependent on the proton gradient and thus mediated by MFS transporters. Further addition of vanadate significantly increased accumulation, revealing a minor contribution from ABC transporters.The *ΔAF-MFS32* mutant (B) exhibited a altered response: CCCP treatment failed to significantly inhibit efflux, and no additional effect was observed with vanadate, suggesting a shift to a non-MFS, non-ABC efflux mechanism.The *ΔAF-MFS35* mutant (C) showed complete loss of efflux function, as indicated by consistently high Rh6G accumulation across all treatments, identifying *AF-MFS35* as an essential component of the efflux pump.

## Discussion

Invasive aspergillosis is often associated with a range of concurrent conditions, such as immunosuppression, multi-organ damage, and chronic drug use [26]. If essential medications exhibit inherent antifungal activity or enhance the efficacy of antifungal agents, they could significantly improve patient outcomes. Proton pump inhibitors (PPIs) are widely used to treat acid-related conditions, including reflux esophagitis, gastric ulcers, duodenal ulcers, and the prevention of ulcer recurrence during treatment with low-dose aspirin or non-steroidal anti-inflammatory drugs (NSAIDs) [27]. Besides inhibiting gastric acid secretion, PPIs are known to induce selective apoptosis and exert cytoprotective effects [28]. The activation of PPIs is most effective under low pH conditions, where they mediate their effects through the early generation of reactive oxygen species (ROS). While previous studies in mammalian systems have shown that NADPH oxidase and p38 MAPK mediate PPI-induced ROS accumulation—leading to lysosomal alkalization and mitochondrial dysfunction [29, 30], these mechanisms may differ in fungi due to distinct cellular physiology. For example, fungal efflux pumps (MFS transporters) and cell wall composition could modulate drug responses differently than in human cells [6, 13]. Thus, our findings in *Aspergillus* highlight the need for fungal-specific mechanistic studies, even when extrapolating from conserved eukaryotic pathways.

In this study, we explored the novel PPI drug, REV, a potassium-competitive acid blocker (P-CAB) that is administered orally and has a different mechanism of action compared to conventional PPIs. REV competitively and reversibly inhibits the potassium-binding site of H^+^/K^+^-ATPase, demonstrating potent inhibitory activity against H^+^/K^+^-ATPase [31]. Both PPIs and P-CABs are used for acid-related diseases, but their clinical effects differ due to variations in their mechanisms of action. While PPIs need to be taken 30–60 min before a meal, P-CABs can be taken at any time. Notably, approximately two-thirds of patients with reflux esophagitis do not achieve symptom control with an initial dose of PPIs, whereas P-CABs are effective from the first dose [32].

In our study, REV alone did not show antifungal activity against the tested *Aspergillus* isolates even at the highest dose. However, when combined with antifungal agents, REV demonstrated synergistic effects and inhibited fungal growth. REV showed synergy with ITR and POS in 20% and 90% of *Aspergillus* species, respectively, with a four-fold reduction in the MIC of both ITR and POS. Conversely, no synergistic effect was observed when REV was combined with VOR. These findings suggest that combinations of REV, particularly with POS, could be promising for treating clinical *Aspergillus* infections.

Our research group has previously demonstrated the synergistic effects of PPIs with triazole antifungals against *Aspergillus in vitro* [8]. This study further confirms that the novel PPI drug, REV, also exhibits synergistic activity when used with antifungal agents. To better understand the mechanism behind the synergy of REV and triazoles, we identified two potential targets from our gene knockout strains of *A. fumigatus*: the major facilitator superfamily (MFS) transporter-related genes, *AF-MFS32* and *AF-MFS35*. MFS transporters are a large group of drug efflux pumps that reduce intracellular drug concentrations, leading to decreased drug sensitivity. Knockout mutants of these genes might reduce drug efflux, resulting in higher intracellular drug concentrations and increased sensitivity to antifungal agents.

The synergistic effects observed between REV and POS may be related to the inhibition of MFS transporter proteins encoded by these genes, *AF-MFS32* and *AF-MFS35*. Given REV clinical use for acid suppression, our findings suggest testable opportunities in immunocompromised patients receiving both P-CABs and azoles, particularly for POS combinations showing 90% synergy *in vitro.*This combination provides a new therapeutic option.

It is well-established that ABC transporters serve as the predominant drivers of rhodamine efflux in many pathogenic fungal species, including *Candida albicans* and *Aspergillus fumigatus* [33, 34]. This established paradigm underscores the significance of our finding that MFS transporters are involved in REV-azole synergy in *A. fumigatus*. Although we cannot entirely rule out complementary activity by ABC transporters, our pharmacological data using CCCP and vanadate clearly demonstrate that proton motive force–dependent MFS transport represents the primary efflux mechanism responsible for Rhodamine 6G extrusion. This fundamental difference in efflux machinery may reflect a unique adaptive evolutionary strategy underlying REV-azole synergy in *A. fumigatus* and could have important implications for its intrinsic resistance profile.

While this study demonstrates promising in vitro synergy between REV and triazoles, several limitations should be noted: (1) The clinical relevance remains uncertain without in vivo validation in animal models of aspergillosis; (2) Pharmacokinetic interactions between REV and POS have not been characterized, particularly regarding potential CYP450-mediated metabolism or tissue penetration; (3) All tested isolates were azole-sensitive, leaving open questions about efficacy against resistant strains with *cyp51A* mutations or other resistance mechanisms. Future studies should address these gaps through murine infection models and expanded testing of resistant clinical isolates.

## Supplementary Information

Supplementary Material 1.

Supplementary Material 2.

Supplementary Material 3.

Supplementary Material 4.

## Data Availability

The datasets generated and analysed during the current study are available in the NCBI’s SRA database repository，accession number：SRR35639660，SRR35639661，SRR35639662，SRR35639663，SRR35639664，SRR35639665，SRR35639666，SRR35639667，SRR35639668，SRR35639669，SRR35639670，SRR35639671；Bio Project ID: PRJNA1334552；link: https://www.ncbi.nlm.nih.gov/bioproject/PRJNA1334552/.
