# Supplementary material for: In vitro combination effects and mechanisms of Revaprazan with Triazole antifungal drugs on Aspergillus
Source: BMC Microbiol. 2025 Nov 5;25:715. doi: 10.1186/s12866-025-04471-w (PMC12587714; doi:10.1186/s12866-025-04471-w)
Supplement: Supplementary file 1 — Supplementary Material 1. [file 12866_2025_4471_MOESM1_ESM.doc]

**Supplementary Materials:**

Table S1 Primer sequences used in this study

| Name  （*AF-MFS32*） | Sequence(5’-3’) | Function |
| --- | --- | --- |
| *AF-MFS32* P1 | ACGAGTGAAACTCCAAATGCATTG | Amplify the upstream |
| *AF-MFS32* P2 | TAGTTCTGTTACCGAGCCGGCCTAGCCACCTCCTGAACAGAC |
| *AF-MFS32* P3 | GCTCTGAACGATATGCTCCAACTCCGTTACAAGACGTGCCCTG | Amplify the downstream |
| *AF-MFS32* P4 | GTATGACTGTAATCGCCGGCATC |
| *AF-MFS32* P5 | GAGGGGACATGGGCAAGTTG | Fusion PCR |
| *AF-MFS32* P6 | TTAGGGTTATGGTACTGCGGCAGA |
| *pyrG-n-F* | CCGGCTCGGTAACAGAACTACCGCAGACAATGCTCTCTATC | *pyrG* Amplify the *pyrG* |
| *pyrG-n-R* | GTTGGAGCATATCGTTCAGAGCAATACCGTTACACATTTCCA |
| Awm-F1 | CCTCGCACAGACAACCAAG | Verify that the filter marker is inserted into the knockout strain |
| *AF-MFS35* P1 | GAAGACTATTCAGATGTGAGTTCTCT | Amplify the upstream |
| *AF-MFS35* P2 | TAGTTCTGTTACCGAGCCGGTATCTACGTTGGTGTTACATGAGGG |
| *AF-MFS35* P3 | GCTCTGAACGATATGCTCCAACGTTGTGTGGGAGGGTTGGATGA | Amplify the downstream |
| *AF-MFS35* P4 | GATTTCAGGTTAAGTGTCGGTGACAA |
| *AF-MFS35* P5 | ATTCCCACGGTGGTAGTCAGAA | Fusion PCR |
| *AF-MFS35* P6 | GTTAATGGGTCCTGGAAGAGATGC |
| Carslan-R4 | AGATGAGGAAGTTGTGCTTTGTC | Verify that the filter marker is inserted into the knockout strain |
