# Supplementary material for: In vitro combination effects and mechanisms of Revaprazan with Triazole antifungal drugs on Aspergillus
Source: BMC Microbiol. 2025 Nov 5;25:715. doi: 10.1186/s12866-025-04471-w (PMC12587714; doi:10.1186/s12866-025-04471-w)
Supplement: Supplementary file 2 — Supplementary Material 2. [file 12866_2025_4471_MOESM2_ESM.doc]

**Supplementary Materials:**

Table S1: Results of MIC and FICI for the combination of REV and antifungal drugs against *Aspergillus* strains

| Strains | MIC alone (µg/mL) | | | | MIC combinations (µg/mL) | | |
| --- | --- | --- | --- | --- | --- | --- | --- |
|  | REV | ITR | VOR | POS | REV/ITR | REV/VOR | REV/POS |
| AF1 | >16 | 2 | 0.5 | 0.5 | 0.25/2(I) | 0.25/1(I) | 8/0.125(S) |
| AF2 | >16 | 4 | 0.5 | 1 | 0.25/4(I) | 0.25/1(I) | 8/0.25(S) |
| AF3 | >16 | 2 | 1 | 1 | 0.25/2(I) | 0.25/1(I) | 4/0.25(S) |
| AF4 | >16 | 2 | 1 | 0.5 | 0.25/2(I) | 0.25/1(I) | 8/0.125(S) |
| AF5 | >16 | 2 | 0.5 | 1 | 0.25/2(I) | 0.25/1(I) | 8/0.25(S) |
| AF6 | >16 | 4 | 1 | 1 | 0.25/4(I) | 0.25/0.5(I) | 8/0.25(S) |
| AF7 | >16 | 2 | 0.5 | 1 | 0.25/2(I) | 0.25/0.5(I) | 8/0.25(S) |
| AF8 | >16 | 4 | 1 | 1 | 0.25/4(I) | 0.25/1(I) | 0.25/1(I) |
| AF9 | >16 | 2 | 0.5 | 0.5 | 8/0.5(S) | 0.25/1(I) | 8/0.125(S) |
| AF10 | >16 | 2 | 0.5 | 0.5 | 4/0.5(S) | 0.25/1(I) | 8/0.125(S) |
| AF11 | >16 | 4 | 0.5 | 1 | 0.25/4(I) | 0.25/0.5(I) | 8/0.25(S) |
| AF12 | >16 | 1 | 0.5 | 0.5 | 0.25/1(I) | 0.25/0.5(I) | 4/0.125(S) |
| AF13 | >16 | 2 | 1 | 0.5 | 8/8(A) | 0.25/1(I) | 0.25/0.5(I) |
| AF14 | >16 | 1 | 0.5 | 0.5 | 0.25/1(I) | 0.25/0.5(I) | 2/0.125(S) |
| AFLA1 | >16 | 2 | 0.5 | 0.5 | 0.25/2(I) | 0.25/0.5(I） | 4/0.25(S) |
| AFLA2 | >16 | 2 | 1 | 0.5 | 0.25/2(I) | 0.25/0.5(I） | 8/0.125(S) |
| AFLA3 | >16 | 2 | 1 | 0.5 | 0.25/2(I) | 0.25/0.5(I） | 8/0.125(S) |
| AFLA4 | >16 | 2 | 1 | 1 | 0.25/2(I) | 0.25/0.5(I） | 8/0.25(S) |
| AFLA5 | >16 | 1 | 1 | 1 | 0.25/1(I) | 0.25/1(I) | 8/0.25(S) |
| AFLA6 | >16 | 1 | 1 | 1 | 0.25/1(I) | 0.25/1(I) | 8/0.25(S) |
| AFLA7 | >16 | 0.5 | 1 | 0.5 | 0.25/0.5（I) | 0.25/1(I) | 2/0.125(S) |
| AFLA8 | >16 | 0.5 | 0.5 | 0.25 | 0.25/0.5(I） | 0.25/0.5(I） | 4/0.0625(S) |
| AFLA9 | >16 | 1 | 0.5 | 0.5 | 0.25/1(I) | 0.25/1(I) | 0.25/0.5(I) |
| AFLA10 | >16 | 1 | 1 | 1 | 0.25/1(I) | 0.25/1(I) | 8/0.25(S) |
| AFLA11 | >16 | 2 | 1 | 1 | 4/0.5(S) | 0.25/0.5(I） | 8/0.25(S) |
| AFLA12 | >16 | 1 | 0.5 | 1 | 2/0.5(I) | 0.25/1(I) | 2/0.25(S) |
| AT1 | >16 | 1 | 0.5 | 1 | 8/0.25(S) | 0.25/0.5(I） | 8/0.25(S) |
| AT2 | >16 | 2 | 0.5 | 0.5 | 8/0.5(S) | 0.25/0.5(I） | 4/0.125(S) |
| AT3 | >16 | 2 | 0.5 | 0.5 | 8/0.5(S) | 0.25/0.5(I） | 8/0.125(S) |
| AT4 | >16 | 1 | 0.5 | 0.5 | 0.25/1(I) | 0.25/0.5(I） | 4/0.125(S) |
| ATCC22019 | >16 | 0.5 | 0.125 | 0.25 | 0.25/0.5(I） | 0.25/0.125（I) | 4/0.0625(S) |
| ATCC204304 | >16 | 2 | 0.5 | 1 | 0.25/2(I) | 0.25/0.5(I) | 8/0.5(I) |

Note：AF, A. fumigatus strains; AFLA, A. flavus; AT, A. terreus; ITR, itraconazole; VOR, voriconazole; POS, posaconazole; REV, revaprazan; S, synergy (FICI ≤ 0.5); I, indifference (no interaction, FICI from >0.5 to ≤4). MICs were the concentrations that achieved 100% growth inhibition.

Table S2 Primer sequences used in this study

| Name  （*AF-MFS32*） | Sequence(5’-3’) | Function |
| --- | --- | --- |
| *AF-MFS32* P1 | ACGAGTGAAACTCCAAATGCATTG | Amplify the upstream |
| *AF-MFS32* P2 | TAGTTCTGTTACCGAGCCGGCCTAGCCACCTCCTGAACAGAC |
| *AF-MFS32* P3 | GCTCTGAACGATATGCTCCAACTCCGTTACAAGACGTGCCCTG | Amplify the downstream |
| *AF-MFS32* P4 | GTATGACTGTAATCGCCGGCATC |
| *AF-MFS32* P5 | GAGGGGACATGGGCAAGTTG | Fusion PCR |
| *AF-MFS32* P6 | TTAGGGTTATGGTACTGCGGCAGA |
| *pyrG-n-F* | CCGGCTCGGTAACAGAACTACCGCAGACAATGCTCTCTATC | *pyrG* Amplify the *pyrG* |
| *pyrG-n-R* | GTTGGAGCATATCGTTCAGAGCAATACCGTTACACATTTCCA |
| Awm-F1 | CCTCGCACAGACAACCAAG | Verify that the filter marker is inserted into the knockout strain |
| *AF-MFS35* P1 | GAAGACTATTCAGATGTGAGTTCTCT | Amplify the upstream |
| *AF-MFS35* P2 | TAGTTCTGTTACCGAGCCGGTATCTACGTTGGTGTTACATGAGGG |
| *AF-MFS35* P3 | GCTCTGAACGATATGCTCCAACGTTGTGTGGGAGGGTTGGATGA | Amplify the downstream |
| *AF-MFS35* P4 | GATTTCAGGTTAAGTGTCGGTGACAA |
| *AF-MFS35* P5 | ATTCCCACGGTGGTAGTCAGAA | Fusion PCR |
| *AF-MFS35* P6 | GTTAATGGGTCCTGGAAGAGATGC |
| Carslan-R4 | AGATGAGGAAGTTGTGCTTTGTC | Verify that the filter marker is inserted into the knockout strain |
| ITS1 | TCCGTAGGTGAACCTGCGG | internal transcribed spacer primer sequence |
| ITS4 | TCCTCCGCTTATTGATATGC |

Table S3 Primer sequences used in this study

| Name | Sequence(5’-3’) | | | | | |
| --- | --- | --- | --- | --- | --- | --- |
|  | P1 | P2 | P3 | P4 | P5 | P6 |
| *ΔAF-MFS13* | AACCAACTAGTTTCCCTGTGCT | TAGTTCTGTTACCGAGCCGGTCCTGCGATTTCAATAAGGGTTGT | GCTCTGAACGATATGCTCCAACGACAAAACGAAATAGAATATGTGTGC | TAAGCATAGCCGAGGCGATGA | GTTTTCAGATCGGCATCACACC | GCTACACAGAGTGCCAAACAG |
| *ΔAF-MFS14* | GGTGTACCCTCACCTGTGGCGGGAG | TAGTTCTGTTACCGAGCCGGATTAGGTTGCCATGCTGGTGAAGAA | GCTCTGAACGATATGCTCCAACACGGTTGATTCCCTTCTCCGATCGA | TGAGTGAGGTTAACTAACTTCGAAA | TCTAATGGCGCGTAGCCTTTATGAT | TAGAGGCTATGACCCGACACGGATT |
| *ΔAF-MFS15* | TCATTGTAGCGTTGGCTTGGGCAT | TAGTTCTGTTACCGAGCCGGACCGTGCAAGGTAAGTTTCTGGTC | GCTCTGAACGATATGCTCCAACCACTACTGTCTAATGCCTTTTGAG | ACCTCGGTGAACTTTGCTCCGGAG | CGGATTCGAGGTCGATCTGAAGGG | GTAAAACCCGAGGCCCCGAACTGC |
| *ΔAF-MFS24* | CAAGGCGTTCGTGACTGGTGAAAT | TAGTTCTGTTACCGAGCCGGGTTGCACGGAATGTCGCAAAGTGG | GCTCTGAACGATATGCTCCAACGCCAGGGTATTCTGCATCATTGTT | TCGATCTGGGGGTTTCCAGTGCAG | GCCGACGAGACCTTCAAGCTCGGT | TTGACAGGGAAGGTCAGGGACTTG |
| *ΔAF-MFS26* | TCGCGGAATGGCGTCAGGCTTGGA | TAGTTCTGTTACCGAGCCGGTTGACTCGCTTATGTCGGGGTGCG | GCTCTGAACGATATGCTCCAACGTGGTCATCCACAGACTTTTCTTT | AACGGGGGGGCGCCAAGGGAGATG | CGACTCCGCCCTATGGGTAGAGAA | GCCATCATGGTAAGAGGTCAGGAG |
| *ΔAF-MFS27* | AGAAGGCTGCACGTCATTAATCTT | TAGTTCTGTTACCGAGCCGGAGCCAAGAAGGTCAACCGCCCGGC | GCTCTGAACGATATGCTCCAACTCCTGATATCTAGAATATGGACTC | TTCTTTTTCACCGCTGTTTATAAA | ACCTGGCGAAGGGAATTACACTAC | CCCTATCTTTAAGGACTGTCGGTG |
| *ΔAF-MFS42* | GGCTGAATTGTTCCTTGGTTGTTC | TAGTTCTGTTACCGAGCCGGGACGGGCGAAGGAGAATGATT | GCTCTGAACGATATGCTCCAACATGACCGTGAGTTTATGTGCGTA | TCCGTCATCTTAATATTTGCCACTT | TGAAACTCTCGGGTACGTTTCGAG | AGAGTAGTACGTCTACGTCCCAT |
| *ΔAF-MFS47* | GCTCCGGCGCATATCTAGATAATT | TAGTTCTGTTACCGAGCCGGGACGAGACAAAAGCAACTGCAGA | GCTCTGAACGATATGCTCCAACTGGCTCTGAAGTAGTACGAAGAAG | AACAAACTATACATCTACCCCTCG | CGTACTTGTCTGTAGGAAAGTAGAT | GCAACCAAGAACGGCAAGTACTA |
| *ΔAF-MFS58* | ACCTGTTGTTACGAACTGCGATGAC | TAGTTCTGTTACCGAGCCGGCCATCAGCTAACACCAACCTCAACA | GCTCTGAACGATATGCTCCAACTATGTATAGTTGCTTTATTTGTTGC | TCCTTATTCACATAGGTCGAATCGA | GATGCAGTGTACAATAATCTGGGTT | AGCTAAAGAAGGAAGAGATCAAAAG |
| *ΔAF-MFS67* | AGTATCTGCGTTGGATTGGCAC | TAGTTCTGTTACCGAGCCGGGTTGCCGACTGCTGAAGTACT | GCTCTGAACGATATGCTCCAACGAGATCTGCCTTGTGAGCCTG | GTCCGATGCCTTCTGGGTC | ACGGTGGATGATTACCTAGCTCA | CTTAGTAGGAAAGGCCAGGACTG |
