# Supplementary material for: In vitro combination effects and mechanisms of Revaprazan with Triazole antifungal drugs on Aspergillus
Source: BMC Microbiol. 2025 Nov 5;25:715. doi: 10.1186/s12866-025-04471-w (PMC12587714; doi:10.1186/s12866-025-04471-w)
Supplement: Supplementary file 3 — Supplementary Material 3. [file 12866_2025_4471_MOESM3_ESM.doc]

**Supplementary Materials:**

Table S1 Primer sequences used in this study

| Name  （*AF-MFS32*） | Sequence(5’-3’) | Function |
| --- | --- | --- |
| *AF-MFS32* P1 | ACGAGTGAAACTCCAAATGCATTG | Amplify the upstream |
| *AF-MFS32* P2 | TAGTTCTGTTACCGAGCCGGCCTAGCCACCTCCTGAACAGAC |
| *AF-MFS32* P3 | GCTCTGAACGATATGCTCCAACTCCGTTACAAGACGTGCCCTG | Amplify the downstream |
| *AF-MFS32* P4 | GTATGACTGTAATCGCCGGCATC |
| *AF-MFS32* P5 | GAGGGGACATGGGCAAGTTG | Fusion PCR |
| *AF-MFS32* P6 | TTAGGGTTATGGTACTGCGGCAGA |
| *pyrG-n-F* | CCGGCTCGGTAACAGAACTACCGCAGACAATGCTCTCTATC | *pyrG* Amplify the *pyrG* |
| *pyrG-n-R* | GTTGGAGCATATCGTTCAGAGCAATACCGTTACACATTTCCA |
| Awm-F1 | CCTCGCACAGACAACCAAG | Verify that the filter marker is inserted into the knockout strain |
| *AF-MFS35* P1 | GAAGACTATTCAGATGTGAGTTCTCT | Amplify the upstream |
| *AF-MFS35* P2 | TAGTTCTGTTACCGAGCCGGTATCTACGTTGGTGTTACATGAGGG |
| *AF-MFS35* P3 | GCTCTGAACGATATGCTCCAACGTTGTGTGGGAGGGTTGGATGA | Amplify the downstream |
| *AF-MFS35* P4 | GATTTCAGGTTAAGTGTCGGTGACAA |
| *AF-MFS35* P5 | ATTCCCACGGTGGTAGTCAGAA | Fusion PCR |
| *AF-MFS35* P6 | GTTAATGGGTCCTGGAAGAGATGC |
| Carslan-R4 | AGATGAGGAAGTTGTGCTTTGTC | Verify that the filter marker is inserted into the knockout strain |
| ITS1 | TCCGTAGGTGAACCTGCGG | internal transcribed spacer primer sequence |
| ITS4 | TCCTCCGCTTATTGATATGC |

Table S2 Primer sequences used in this study

| Name | Sequence(5’-3’) | | | | | |
| --- | --- | --- | --- | --- | --- | --- |
|  | P1 | P2 | P3 | P4 | P5 | P6 |
| *ΔAF-MFS13* | AACCAACTAGTTTCCCTGTGCT | TAGTTCTGTTACCGAGCCGGTCCTGCGATTTCAATAAGGGTTGT | GCTCTGAACGATATGCTCCAACGACAAAACGAAATAGAATATGTGTGC | TAAGCATAGCCGAGGCGATGA | GTTTTCAGATCGGCATCACACC | GCTACACAGAGTGCCAAACAG |
| *ΔAF-MFS14* | GGTGTACCCTCACCTGTGGCGGGAG | TAGTTCTGTTACCGAGCCGGATTAGGTTGCCATGCTGGTGAAGAA | GCTCTGAACGATATGCTCCAACACGGTTGATTCCCTTCTCCGATCGA | TGAGTGAGGTTAACTAACTTCGAAA | TCTAATGGCGCGTAGCCTTTATGAT | TAGAGGCTATGACCCGACACGGATT |
| *ΔAF-MFS15* | TCATTGTAGCGTTGGCTTGGGCAT | TAGTTCTGTTACCGAGCCGGACCGTGCAAGGTAAGTTTCTGGTC | GCTCTGAACGATATGCTCCAACCACTACTGTCTAATGCCTTTTGAG | ACCTCGGTGAACTTTGCTCCGGAG | CGGATTCGAGGTCGATCTGAAGGG | GTAAAACCCGAGGCCCCGAACTGC |
| *ΔAF-MFS24* | CAAGGCGTTCGTGACTGGTGAAAT | TAGTTCTGTTACCGAGCCGGGTTGCACGGAATGTCGCAAAGTGG | GCTCTGAACGATATGCTCCAACGCCAGGGTATTCTGCATCATTGTT | TCGATCTGGGGGTTTCCAGTGCAG | GCCGACGAGACCTTCAAGCTCGGT | TTGACAGGGAAGGTCAGGGACTTG |
| *ΔAF-MFS26* | TCGCGGAATGGCGTCAGGCTTGGA | TAGTTCTGTTACCGAGCCGGTTGACTCGCTTATGTCGGGGTGCG | GCTCTGAACGATATGCTCCAACGTGGTCATCCACAGACTTTTCTTT | AACGGGGGGGCGCCAAGGGAGATG | CGACTCCGCCCTATGGGTAGAGAA | GCCATCATGGTAAGAGGTCAGGAG |
| *ΔAF-MFS27* | AGAAGGCTGCACGTCATTAATCTT | TAGTTCTGTTACCGAGCCGGAGCCAAGAAGGTCAACCGCCCGGC | GCTCTGAACGATATGCTCCAACTCCTGATATCTAGAATATGGACTC | TTCTTTTTCACCGCTGTTTATAAA | ACCTGGCGAAGGGAATTACACTAC | CCCTATCTTTAAGGACTGTCGGTG |
| *ΔAF-MFS42* | GGCTGAATTGTTCCTTGGTTGTTC | TAGTTCTGTTACCGAGCCGGGACGGGCGAAGGAGAATGATT | GCTCTGAACGATATGCTCCAACATGACCGTGAGTTTATGTGCGTA | TCCGTCATCTTAATATTTGCCACTT | TGAAACTCTCGGGTACGTTTCGAG | AGAGTAGTACGTCTACGTCCCAT |
| *ΔAF-MFS47* | GCTCCGGCGCATATCTAGATAATT | TAGTTCTGTTACCGAGCCGGGACGAGACAAAAGCAACTGCAGA | GCTCTGAACGATATGCTCCAACTGGCTCTGAAGTAGTACGAAGAAG | AACAAACTATACATCTACCCCTCG | CGTACTTGTCTGTAGGAAAGTAGAT | GCAACCAAGAACGGCAAGTACTA |
| *ΔAF-MFS58* | ACCTGTTGTTACGAACTGCGATGAC | TAGTTCTGTTACCGAGCCGGCCATCAGCTAACACCAACCTCAACA | GCTCTGAACGATATGCTCCAACTATGTATAGTTGCTTTATTTGTTGC | TCCTTATTCACATAGGTCGAATCGA | GATGCAGTGTACAATAATCTGGGTT | AGCTAAAGAAGGAAGAGATCAAAAG |
| *ΔAF-MFS67* | AGTATCTGCGTTGGATTGGCAC | TAGTTCTGTTACCGAGCCGGGTTGCCGACTGCTGAAGTACT | GCTCTGAACGATATGCTCCAACGAGATCTGCCTTGTGAGCCTG | GTCCGATGCCTTCTGGGTC | ACGGTGGATGATTACCTAGCTCA | CTTAGTAGGAAAGGCCAGGACTG |

Table S3 Data statistical results by the disk diffusion method

| Strain | Diameter(cm) | | | | | | | | | | | |
| --- | --- | --- | --- | --- | --- | --- | --- | --- | --- | --- | --- | --- |
|  | Saline | | | REV | | | POS | | | REV+POS | | |
| *ΔAF-MFS27* | 0 | 0 | 0 | 0 | 0 | 0 | 2.4 | 2.4 | 2.5 | 2.5 | 2.4 | 2.4 |
| *ΔAF-MFS32* | 0 | 0 | 0 | 0 | 0 | 0 | 2.8 | 2.9 | 2.8 | 2.4 | 2.3 | 2.4 |
| *ΔAF-MFS35* | 0 | 0 | 0 | 0 | 0 | 0 | 2.8 | 2.7 | 2.8 | 2.4 | 2.3 | 2.2 |
| WT | 0 | 0 | 0 | 0 | 0 | 0 | 2.7 | 2.7 | 2.6 | 2.6 | 2.6 | 2.6 |
