# Supplementary figures and images for: In vitro combination effects and mechanisms of Revaprazan with Triazole antifungal drugs on Aspergillus
Source: BMC Microbiol. 2025 Nov 5;25:715. doi: 10.1186/s12866-025-04471-w (PMC12587714; doi:10.1186/s12866-025-04471-w)

Figure 2A

A


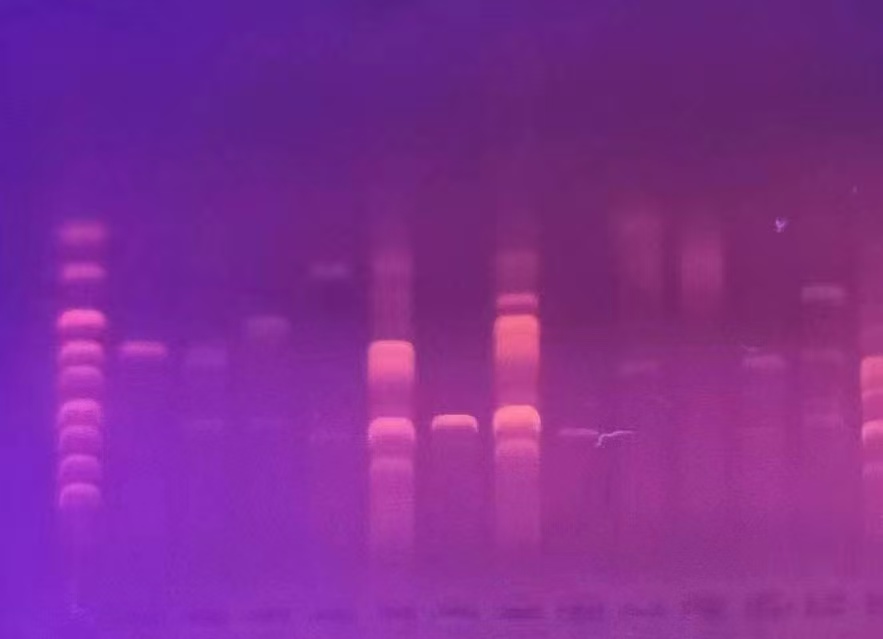

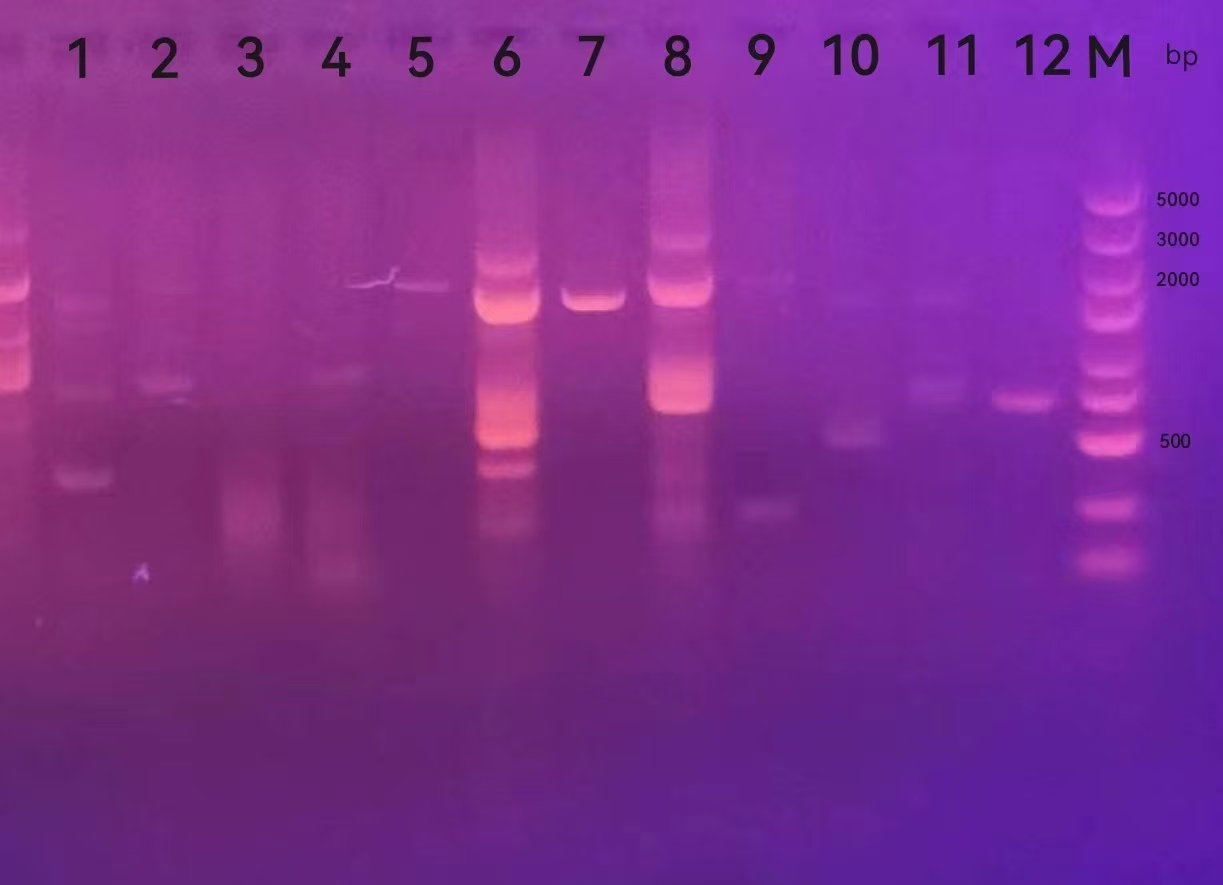


Figure 2B


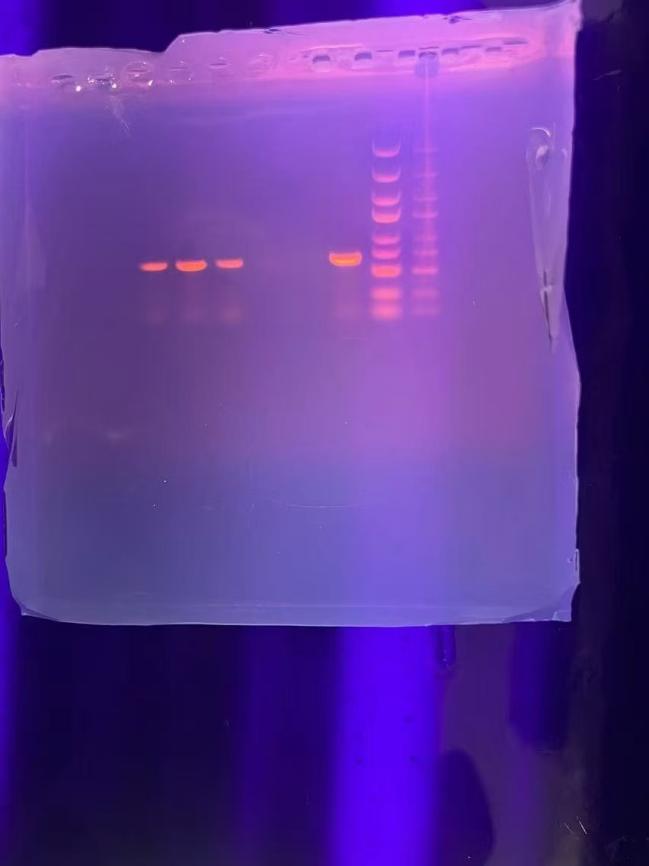


Figure 1


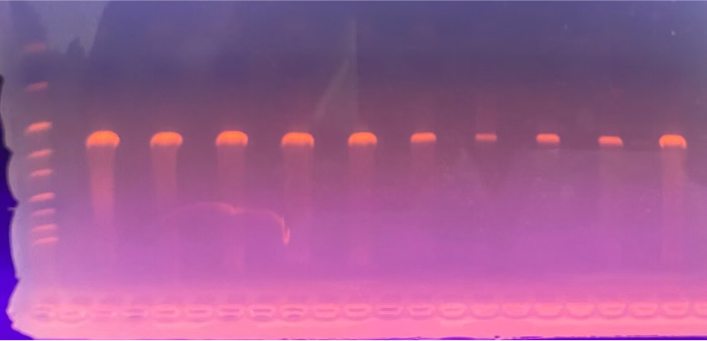

Supplement: Supplementary file 4 — Supplementary Material 4. [file 12866_2025_4471_MOESM4_ESM.docx]
